# COVID-19 in Egypt: Uncovered figures or a different situation?

**DOI:** 10.7189/jogh.10.010368

**Published:** 2020-06

**Authors:** Mohammed A Medhat, Mohamed El Kassas

**Affiliations:** 1 Tropical Medicine and Gastroenterology Department, Faculty of Medicine, Assiut University, Cairo, Egypt; 2 Endemic Medicine Department, Faculty of Medicine, Helwan University, Cairo, Egypt

The newly discovered Coronavirus, recently named SARS-CoV-2, emerged from The newly discovered Coronavirus, recently named SARS-CoV-2, which emerged from Wuhan, China, resulted in a pandemic public health emergencies. As of April 19, 2020, Egypt has reported 3144 cases of COVID-19 and 239 deaths. However, some reports claimed that these figures are underrated, especially with the documentation of several exported cases from Egypt to other countries, and that the burden of COVID-19 infection in Egypt, therefore, might be considerably more significant than reported. Meanwhile, Egypt has established a convincing model of care in order to combat the COVID-19 epidemic under the supervision of the World Health Organization. In this report, we tried to highlight the situation of COVID-19 infection in Egypt, the possible causes of the relatively lower rates of infection in the country like high temperature, humidity, early use of BCG vaccination, and possibly a different viral subtype. This is besides detailing the state and preventive measures taken to face this epidemic by the country.

The newly discovered Coronavirus, recently named SARS-CoV-2 (COVID-19), emerged from Wuhan, China, resulting in a pandemic public health emergencies. Recent epidemiological data shows that there are more than 2 million people were infected with SARS-CoV-2 with a mortality rate of >6.7% [1]. In an earlier report, Egypt had the highest risk of SARS-CoV-2 infection importation among African countries [2]. On February 14, 2020, Egypt announced the first case of COVID-19 in Africa and needed about three months to record 10 000 cases, which was almost double the time that Italy and the USA took to reach the same number of cases. Tuite et al. proposed an estimation of the outbreak size of 19310 cases of COVID-19 (95% confidence interval (CI) = 6270-45 070) in Egypt based on diagnosed cases coming from Egypt in different countries [3]. This data does not coop with what is announced by the Egyptian Ministry of Health (MOH), and thus a big question was aroused; Does Egypt have uncovered data about the real number of COVID-19 cases?

## EGYPTIAN STATE EFFORTS TO CONTROL COVID-19 INFECTION

Since the announcement of the first case of the SARS-CoV-2 case in Egypt, the government started multidisciplinary national coordination between different ministries. In February, they decided that there is no need to stop close airport travels, schools, or universities at this stage. Instead, Egypt started to examine all tourists coming to Egypt and to trace them for the development of fever or respiratory symptoms after arrival. Egypt discovered cases of COVID-19 in a cruise ship in Luxor, and subsequently, the authorities prevented the onboard passengers and the crew from leaving the ship and sent a medical team to take care of them. On March 8, Egypt announced that a 60 year-old German tourist died in the touristic town Hurghada, Egypt, and this was the first German fatality from the virus. The escalation in the number of cases by the middle of March to be >100 cases prompted the government to take more strict arrangements. Egypt suspended schools and universities for one month and encouraged electronic distance learning. Also, Egypt imposed a curfew from 7 pm until 6 am, during which all shops and markets are closed from 5 pm to 6 pm and are subjected to a complete shutdown on Fridays and Saturdays. Besides, all means of public and private transportation are suspended during curfew hours. Flight into or out of Egypt were also suspended in an attempt to reduce the risk of importing infection. All sports and many social activities were also banned to prevent the spread of COVID-19.

## HEALTH MEASURES TO CONTROL INFECTION

Egypt abandoned individual hospitals in every governate to be assigned as quarantine hospitals for COVID-19 patients. Egyptian MOH decided to relay on a medical team composed of different specialties (Pulmonology, Internal Medicine, Tropical Medicine, Infectious Diseases, Intensive care, Radiology, Clinical Pathology, Clinical Pharmacy, and Infection control) to take care of the inpatients in these quarantine hospitals. Each medical team stays in the hospital for 14 days, then all team members should be tested for SARS-CoV-2 through nasopharyngeal swabs, and infection negative team members are released for home self-isolation for another 14 days. Positive swab doctors (if any), are to stay in the quarantine hospital to get medical care.

Egyptian MOH issued a standardized guide for the diagnosis and management of COVID-19. According to this protocol, patients with COVID-19 infection are classified on clinical bases into mild, moderate, severe, and critical cases. Mild cases are patients in whom clinical symptoms are mild, with no signs of pneumonia in chest imaging. Moderate cases are those complaining of COVID-19 symptoms such as fever or manifestations of chest disease, or imaging results indicative of pneumonia. Severe cases are patients who meet any of the following criteria; (a) respiratory rate >30 breaths/min, (b) oxygen saturations <93% at a rest state, (c) arterial partial pressure of oxygen (PaO_2_)/ Fraction of inspired oxygen (FiO_2_)<300 mm Hg, or (d) patients with more than 50% lesions progression within 24 to 48 hours in lung imaging. Finally, critical patients are those who are meeting any of the following criteria; (a) occurrence of respiratory failure requiring mechanical ventilation, or (b) the presence of shock; other organ failures that require monitoring and treatment in the ICU. The setup of quarantine hospitals is prepared to match this protocol.

**Figure:**
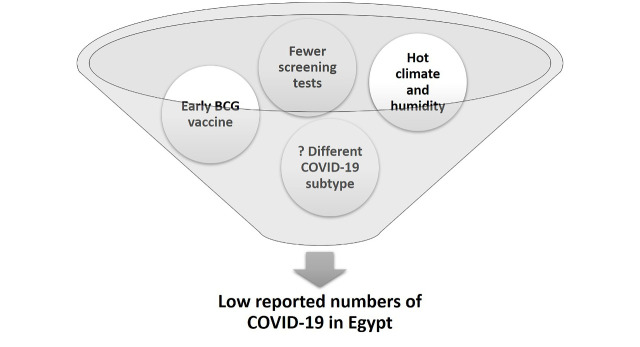
Photo: Low reported numbers of COVID-19 in Egypt (created by the authors, and used with permission).

## FACTORS THAT MAY CONTRIBUTE TO LOWER THE RATE OF INFECTION IN EGYPT

The relatively low rates of newly reported COVID-19 cases in Egypt, and the modest cumulative number of cases compared to other countries could be attributed to some country-specific factors:

## TEMPERATURE AND HUMIDITY

Previous studies about the outbreak of severe acute respiratory syndrome (SARS) declared a decline in the infection rate of the disease with the warming of weather. A Korean study found that low daily temperature and low relative humidity were associated with a significant increase in the incidence of influenza [4]. Absolute humidity also had significant correlations with influenza viral survival and transmission rates [5]. Additionally, some studies reported that meteorological factors could significantly affect COVID-19 rates of infection. Moreover, the doubling time of COVID-19 positively was found to be correlated with temperature and inversely with humidity, implying that a decline in the infection progression rate will occur in spring and summer, as a 20°C increase in temperature is expected to lag the doubling time 1.8 days [6].

Egypt is known to have a hot desert climate which is generally arid all over the country. Moreover, the temperature is extremely high during the summer months, especially in Upper Egypt. By comparing the average temperature during March 2020 in Cairo and other worldwide cities with a high incidence of COVID-19 infection (Milan, Albacete, New York, and Wuhan), noticeable differences are present with higher temperatures in Cairo. Moreover, Cairo had lower humidity during the same period (**Table 1**) [7]. What could support this theory is that the rate of infection is lower in Upper Egypt, where the higher temperature climate is encountered compared to other areas of country. Probably, the climate may have a significant impact on lowering the rate of COVID-19 infection in Egypt.

**Table 1:** Comparison between the weather in Cairo, Milan, Albacete, New York, and Wuhan during March 2020

	Cairo	Milan	Albacete	New York	Wuhan
**Daytime temperature (°C)**	24	14.3	16.3	9.8	14.8
**Nightly temperature**	11	3.8	3.3	0.8	6
**Average temperature**	17.5		9.8	5.3	10.4
**Daily sunshine (hours)**	9.1	4.9	7	7.5	4.2
**Average rainfall (mm)**	2.2	265	27	101.8	95.1
**Rainy days**	2	7	5	11	15
**Humidity (%)**	54	71	63	60	79

## BCG VACCINE USE

Bacille Calmette-Guérin (BCG) is a live attenuated vaccine against *Mycobacterium bovis* (*M.* *bovis*). BCG vaccine has been shown to produce non-specific immune effects leading to enhanced response against other non-mycobacterial organisms [8]. Trained immunity is a term called to describe a proposed metabolic and epigenetic changes following BCG vaccination resulting in an advancement of genetic regions encoding for pro-inflammatory cytokines. BCG vaccination boosts the release of pro-inflammatory cytokines, specifically IL-1β, which has a potent antiviral effect [9]. Some recent studies claimed that BCG vaccination might play a role in reducing the rate of COVID-19, and rates of its related morbidity and mortality. In a recent report, countries that had never implemented universal vaccination policies (Italy, USA, and Nederland) have higher rates of COVID-19 infection and increased mortality. Countries that applied the universal BCG vaccination strategy late in the 20^th^ century, like Iran, were also noticed to have increased rates of infection and mortality, which might be due to unprotected older population [8]. Egypt included BCG vaccination in compulsory vaccination program early since 1974. According to this theory, the relatively low rates of COVID-19 infections and mortalities in Egypt may be partially attributed to the early intake of BCG vaccination in the country. However, no available data up till now could support such a relation or explain why some countries which have never provide BCG vaccination for the population has a low rate of infection.

## PREVALENT COVID-19 SUBTYPE

Two significant subtypes of SARS-CoV-2 are detected based on two Single nucleotide polymorphism (SNP). The L type was found to be derived from the S type, but L ( ~ 70%) is more prevalent than S ( ~ 30%). This suggests that L has a higher transmission rate than the S type [10]. Clinical picture and mortality rates could be correlated with the prevalent type of COVID-19. Up to date, no data are available about the epigenetics of the type of COVID-19 in Egypt. The relatively low number of cases and low case fatality in the country may suggest a less aggressive type of COVID-19.

## THE EGYPTIAN SITUATION: A FUTURE POLL

The rapidly varying situation of the disease across the globe could easily make this report unsound at a particular stage, yet hopefully soon, and once the COVID-19 universal disaster is arrested, many facts for the future will be clarified. For the time being, we call for adhering to more conservative measures till reaching a downsloping curve for the number of reported cases in the country.
